# miR-21 Expression in Cancer Cells may Not Predict Resistance to Adjuvant Trastuzumab in Primary Breast Cancer

**DOI:** 10.3389/fonc.2014.00207

**Published:** 2014-08-15

**Authors:** Boye Schnack Nielsen, Eva Balslev, Tim Svenstrup Poulsen, Dorte Nielsen, Trine Møller, Christiane Ehlers Mortensen, Kim Holmstrøm, Estrid Høgdall

**Affiliations:** ^1^Bioneer A/S, Hørsholm, Denmark; ^2^Department of Pathology, Herlev Hospital, University of Copenhagen, Herlev, Denmark; ^3^Department of Oncology, Herlev Hospital, University of Copenhagen, Herlev, Denmark

**Keywords:** biomarker, breast cancer, HER2, miR-21, prediction, response, trastuzumab

## Abstract

Trastuzumab is established as standard care for patients with HER2-positive breast cancer both in the adjuvant and metastatic setting. However, 50% of the patients do not respond to the trastuzumab therapy, and therefore new predictive biomarkers are highly warranted. MicroRNAs (miRs) constitute a new group of biomarkers and their cellular expression can be determined in tumor samples by *in situ* hybridization (ISH) analysis. miR-21 is highly prevalent and up-regulated in breast cancer and has been linked to drug resistance in clinical and *in vitro* settings. To determine expression patterns of miR-21 in high-grade breast cancers, we examined miR-21 expression in 22 HER2-positive tumors and 15 HER2-negative high-grade tumors by ISH. The histological examination indicated that patient samples could be divided into three major expression patterns: miR-21 predominantly in tumor stroma, predominantly in cancer cells, or in both stromal and cancer cells. There was no obvious difference between the HER2-positive and HER2-negative tumors in terms of the miR-21 expression patterns and intensities. To explore the possibility that miR-21 expression levels and/or cellular localization could predict resistance to adjuvant trastuzumab in HER2-positive breast cancer patients, we analyzed additional 16 HER2-positive tumors from patients who were treated with trastuzumab in the adjuvant setting. Eight of the 16 patients showed clinical recurrence and were considered resistant. Examination of the miR-21 expression patterns and intensities revealed no association between the miR-21 scores in the cancer cell population (*p* = 0.69) or the stromal cells population (*p* = 0.13) and recurrent disease after adjuvant trastuzumab. Thus, our findings show that elevated miR-21 expression does not predict resistance to adjuvant trastuzumab.

## Introduction

The human epidermal growth factor receptor-2 (HER2, NEU, and c-ERBB-2) is a cell surface receptor tyrosine kinase that is strongly up-regulated in more than 15% of all breast cancers (1), and breast cancer patients with HER2 overexpressing tumors have poor prognosis (2, 3). HER2-directed therapy using the humanized monoclonal antibody, trastuzumab in combination with conventional chemotherapy, improves overall survival in patients with HER2-positive breast cancers (4). However, only half of the HER2-positive breast cancer patients respond to the HER2-directed therapy (5). The lack of therapeutic efficacy and high cost of the therapeutic agent urges identification and development of predictive markers as companion diagnostics. So far, no clinically validated predictive markers for response to trastuzumab have been reported.

MicroRNAs (miRs) are short non-coding RNAs that regulate protein synthesis at the post-transcriptional level by binding to the 3’UTR of mRNAs causing mRNA destabilization or degradation. miRs can be measured in tissues and body fluids by *in situ* hybridization (ISH), qPCR, and microarray or high-throughput sequencing, and as such, miRs constitute a relatively novel group of biomarkers. One of the most abundant miRs in solid tumors is miR-21. miR-21 expression is highly increased in malignant tumors, including breast, colon, lung, and brain cancer (6, 7), and high expression levels are associated with poor prognosis (8–11). Solid tumors, including breast cancers, are complex tissues consisting of the malignant epithelial cells and a surrounding stroma consisting of fibroblasts, inflammatory cells, and vascular cells. ISH studies of tissue from breast, colon, brain, pancreas, and esophagus cancer (10–14) have shown that miR-21 is predominantly expressed in the stromal cells; however, subpopulations of miR-21 positive cancer cells are also reported (10, 11, 15).

Some miRs are found to be indicators of drug resistance and some even to confer drug resistance to a variety of cancer drugs (16–18). miR-21 is one the most studied and has been found to confer drug resistance to trastuzumab (19), 5-fluorouracil (20, 21), doxorubicin (22, 23), cisplatin (24, 25), and paclitaxel (26). Gong et al. (19) reported that high miR-21 levels in breast cancer biopsies before and after neoadjuvant trastuzumab were associated with trastuzumab resistance, and that blocking the action of miR-21 re-sensitized resistant breast cancer cell lines to trastuzumab (19). The authors suggested that miR-21 may function through the tumor suppressor phosphatase and tensin homolog (PTEN), a well-described miR-21 target protein (27–29), which has been reported to be a potential predictor for trastuzumab resistance (30). Thus, miR-21 may play an important role in multi-drug resistance mechanisms.

Recent studies have indicated that drug resistance in cancer therapy is not only related to the malignant cancer cells, but also to cells in the stromal compartment (31–34). For example, in the study by Alkhateeb et al. (34), high levels of inflammation markers measured in serum from HER2-positive breast cancer patients were associated with poor response to trastuzumab-containing therapy. Thus, molecular biomarkers derived from the breast cancer stroma should also be considered in the search for novel predictive biomarkers.

In this paper, we addressed whether miR-21 ISH analysis in primary breast cancers can help to predict trastuzumab resistance in HER2-positive breast cancer patients treated with trastuzumab in the adjuvant setting, and whether the miR-21 expression pattern correlated with HER2 status or other known clinical markers.

## Materials and Methods

### Patients

Tissue samples were separated into two sample groups, here named reference group and study group (see also Table 1). The reference group included 36 breast cancer samples from patients diagnosed 1999–2009 with high-grade invasive ductal carcinoma (IDC). Among them, 21 cases were HER2-positive and 15 cases were HER2-negative. The study group included samples from 16 HER2-positive ethnic Danish breast cancers patients. These patients were diagnosed from 2005 to 2008 and had their primary tumor surgically removed and received adjuvant trastuzumab (trastuzumab/Herceptin was obtained from Roche). After surgery, the patients received adjuvant chemotherapy, irradiation, and anti-hormonal treatment according to national standards, and eventually trastuzumab. All patients received trastuzumab every third week for one year. None of the patients had been treated for breast cancer previously. Eight patients developed recurrent disease with distant metastases after 5–8 years of follow up. In the present study, we considered these patients as resistant to trastuzumab. Patients without recurrence were considered sensitive. All samples were obtained from the local tissue bank (Herlev Hospital, Copenhagen, Denmark) as formalin-fixed paraffin-embedded (FFPE, here fixed within 1 h of surgery by 24–72 h incubation in formalin at room temperature). Tumor grade was based on HE stained sections. The study was performed in accordance with permission given by The Regional Scientific-Ethical Committee for Copenhagen, Denmark (J. nr. H-3-2014-010).

**Table 1:** **Patients included in this study**.

	*n*	Age (range)	Diagnosis	HER2	HER2
			year	neg (*n*)	pos (*n*)
Reference group	36	62 (36–92)	1999–2009	15	21
Study group	16	51 (33–68)	2005–2008	0	16

*Age range and year range of diagnosis are indicated for the reference group and study group*.

### Immunohistochemistry

Immunoperoxidase staining for clinical biomarkers included staining for ER (mAb clone SP1, Dako, Glostrup, Denmark), PR (mAb clone PqR636, Dako), P53 (mAb clone DO7, Dako), Ki67 (mAb clone Mib1, Dako), and HER2 (mAb clone 4B5, Roche). For ER (RTU), PR (mAb diluted 1:100), P53 (mAb diluted 1:200), and Ki67 (mAb diluted 1:200), 3–5 μm tissue sections were pretreated using DakoLink and stained in a Dako immunostainer, whereas HER2 staining was performed using a Ventana instrument (Ventana) according to the manufacturers guidelines. Scores of ER, PR, p53, and Ki67 were the percentage of positive cancer cells. The IHC scores of HER2 were performed in accordance with the HercepTest™ Interpretation Manual – Breast (Dako, Glostrup, Denmark).

### HER2 FISH

Cases that scored 2 in HER2 immunoperoxidase staining were additionally tested by FISH analysis using the HER2 FISH pharmDx™ Kit-K331 (Dako, Glostrup, Denmark) according to the manufacturers’ instructions.

### MicroRNA-21 *in situ* hybridization and scoring

**Figure 1:**
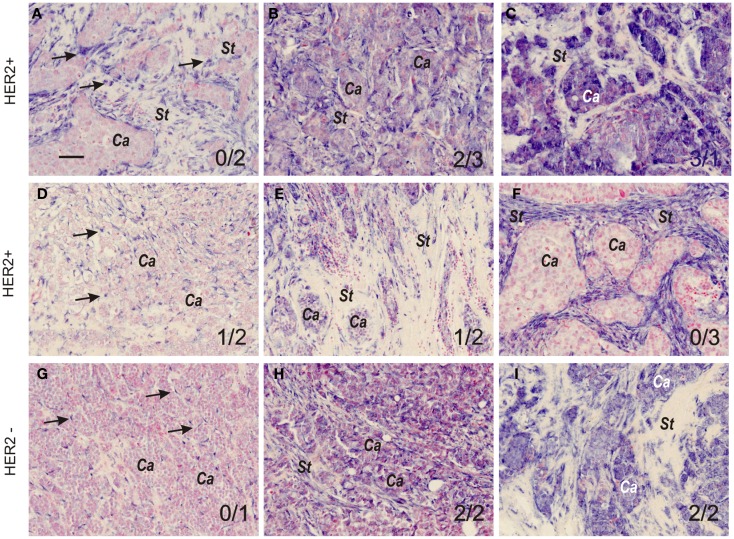
**MicroRNA-21 expression patterns and scoring in grade 3 breast cancers**. Representative miR-21 ISH expression patterns and intensities in HER2-positive **(A–F)** and HER2-negative tumors **(G–I)**. The miR-21 ISH signal (dark blue staining, examples indicated by arrows in **(A)**) is seen exclusively in stromal cells **(A,D,F**, and **G)**, or in both stromal cells and cancer cells **(B,C,E,H**, and **I)**. Stromal and cancer cell tissue compartments are indicated by *St* and *Ca*, respectively. In **(D,G**, and **H)**, arrows indicate miR-21 positive stromal cells. The variations in miR-21 ISH signal intensity and prevalence of positive cells were scored 0, 1, 2, or 3 by two observers (see Material and Methods section) in both cancer and stromal cells. The miR-21^CC^ and miR-21^St^ scores are indicated (miR-21^CC^/miR-21^St^). All sections were counter stained with nuclear fast red. The examples are from tumors in the reference group and are representative for the individual tumors. Bar: 50 μm.

Automated miR-21 ISH was performed on a Tecan Genepaint instrument (Tecan, Switzerland) essentially as described previously (10). In brief, deparaffinized sections were treated with proteinase-K (25 μg/mL for 8 min at 37ŶC). Double-FAM-labeled miR-21 and scrambled LNA™ probes (Exiqon, Vedbæk, Denmark) were incubated at 30 nM for 1 h in Exiqon hybridization buffer (Exiqon) at 57ŶC. The probes were detected with alkaline-phosphatase conjugated anti-FAM (1:800, Roche) for 30 min at 30ŶC and then incubated with freshly prepared NBT-BCIP substrate containing 0.2 nM levamisole for 1 h at 30ŶC. For scoring of the miR-21 ISH staining, we obtained digital whole slides using a Hamamatsu scanner (20× objective). The miR-21 staining was scored at the level of cellular tissue compartments, stromal vs. cancer cells, and at the level of staining intensity/density (0, 1, 2, or 3). Score 0, indicated no staining or similar staining as background level, score 1 indicated staining in a subset of cells that could be weak or intense (up to 10%), score 2 indicated staining in a larger subset of cells (10–50% of the cells) that could be weak or intense, and score 3 indicated intense staining in virtually all cells of the tissue compartment (examples of miR-21 scores are shown in Figure 1). Scoring was performed independently blinded by two observers (Eva Balslev and Boye Schnack Nielsen). If the difference between scores was more than one, a consensus score was determined. The averages of the two scores are presented.

### MicroRNA-21/HER2 double staining

MicroRNA-21 ISH combined with immunohistochemical staining for HER2 was performed essentially as described (35). After proteinase-K treatment, sections were hybridized with 20 nM miR-21 LNA probe for 1 h, and the probe was detected with peroxidase-conjugated anti-FAM (Roche) followed by incubation tyramine-signal-amplification (TSA)-Cy5 substrate for 5 min at room temperature. Polyclonal rabbit anti-ErbB-2 (ab2428, AbCam, Cambridge, UK) was incubated at room temperature and detected with Cy3-conjugated goat anti rabbit (Jackson ImmunoResearch, West Grove, PA, USA). Slides were mounted with Antifade Gold with DAPI (Invitrogen).

### Statistics

Spearman’s rank correlation analyses of miR-21 scores and known clinical parameters were conducted to obtain Spearman rank correlation coefficients, *r*. The differences in miR-21 scores between HER2-positive and HER2-negative, and trastuzumab-sensitive and -resistant patient groups were evaluated using the non-parametric Mann–Whitney *U* test. Statistical significance was considered at the 5% probability level (*p* < 0.05). All statistical analyses and calculations were performed with GraphPad Prism 5 (GraphPad Sofware, La Jolla, CA, USA).

## Results

### Patients

The patient material is briefly described in Table 1. The patient samples in the reference group were used to delineate miR-21 expression patterns and intensities in high-grade breast cancers and to establish a miR-21 scoring system. The miR-21 scores were compared with known clinical parameters, including the HER2 status. The samples in the study group were used to evaluate the miR-21 expression patterns and intensities, to obtain miR-21 scores for correlation with recurrence of disease after adjuvant trastuzumab.

### MicroRNA-21 expression patterns and scoring

MicroRNA-21 ISH was performed in parallel on the 36 samples on an automated platform. miR-21 ISH signal was seen in all cases with variation both in staining intensity and in localization. Among the 36 tumors, all showed staining in tumor stromal cells, mostly fibroblasts, but probably also inflammatory and endothelial cells were positive. The stromal miR-21 staining varied from confined focal staining, with most intense staining in the fibroblastic cells surrounding islands of cancer cells, to staining involving most of or the whole tumor stroma. Using a semiquantitative approach, we scored the miR-21 stroma staining (miR-21^st^) gradually 1, 2, or 3 as described in Material and Methods section (examples in Figure 1). miR-21 ISH signal was also prevalent in cancer cells (miR-21^CC^) in 23 of the cases, and the positive reaction was scored 0, 1, 2, or 3 in terms of staining intensity (examples in Figure 1). In general, miR-21 ISH signal was equally intense in all stained tumor cells in the same tumor, however, some variations were observed. Most dramatic variation was seen in two cases (both HER2-negative) in which one tumor cell compartment showed strong staining, whereas an adjacent tumor cell compartment showed virtually no staining (data not shown). In these two cases the scores presented are those with highest staining intensity. Intensely stained myoepithelial cells surrounding foci of carcinoma *in situ* (CIS) were seen in 8 of the 36 cases (data not shown). No ISH signal was obtained with the scramble probe in any of the samples. Additional specificity analyses of the miR-21 ISH probe in breast cancer samples have been performed previously (12).

### MicroRNA-21 localization and HER2 status and other clinical parameters

**Figure 2:**
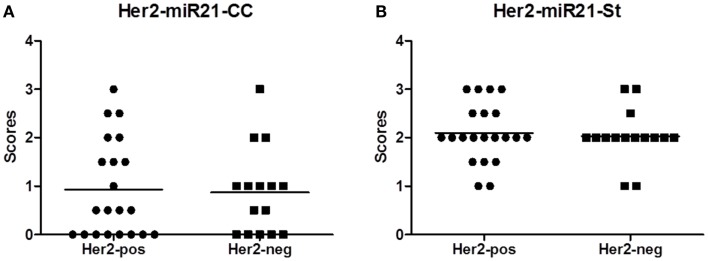
**MicroRNA (miR)-21 scores in the HER2 reference group**. The miR-21 scoring was performed as described in Material and Methods section, here presented as average scores of two observers: miR-21^CC^ **(A)** and miR-21^St^ **(B)**.

There was no obvious difference in the miR-21^CC^ expression pattern or miR-21^St^ expression pattern when comparing HER2-positive with HER2-negative cases (Figure 2), suggesting that the miR-21 expression pattern is independent of the HER2 status. A series of additional molecular parameters were obtained from the cohort by immunohistochemistry: ER, PR, Ki67, and p53 and then correlated with the miR-21 scores (Table 2). In HER2-negative tumors, we noted a significant positive correlation (*p* = 0.04) between miR-21^CC^ and elevated PR. Otherwise, none of these parameters showed significant correlation with the two miR-21 scores, miR-21^CC^ and miR-21^St^. We have previously reported that increased stromal miR-21 levels in grade 1 and 2 lesions are associated with increased cancer cell proliferation as measured by the Ki67 index (12). We did not see a similar correlation in these grade 3 lesions.

**Table 2:** **Correlation analyses**.

		*n*	miR-21^St^	ER	PR	Ki67	P53
miR-21^CC^	HER2 pos	21	−0.31	−0.07	0.03	−0.24	0.03
	HER2 neg	15	−0.14	0.38	0.56*	−0.04	0.35
	All	36	−0.22	0.07	0.22	−0.15	0.15

		***n***	**miR-21^CC^**	**ER**	**PR**	**Ki67**	**P53**

miR-21^St^	HER2 pos	21	−0.31	0.11	0.19	−0.19	−0.24
	HER2 neg	15	−0.14	−0.26	0.13	−0.17	−0.37
	All	36	−0.22	−0.07	0.14	−0.22	−0.26

Spearman correlation analyses of miR-21 scores (cancer cell expression, miR-21^CC^ and stromal expression, miR-21^St^) and available clinical parameters here separated into HER2-negative and positive tumors (Spearman correlations, *p < 0.05).

### MicroRNA-21 co-localizes with HER2

**Figure 3:**
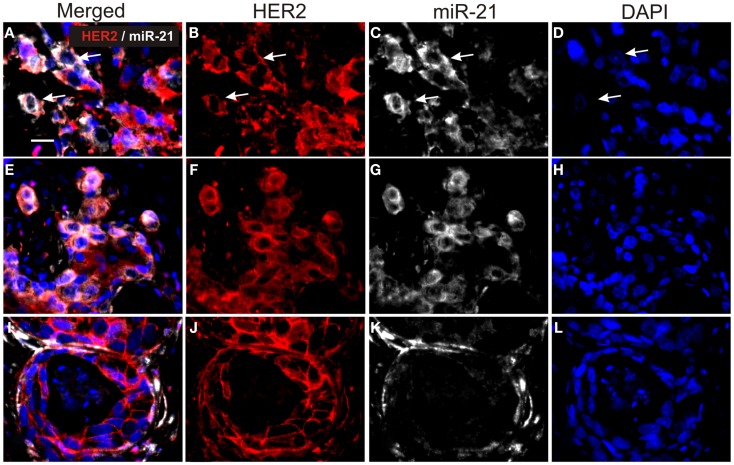
**Double staining for miR-21 and HER2 in HER2-positive breast cancers**. Tissue sections were processed first for miR-21 ISH and subsequently for HER2 IHC. The figure shows three cases **(A–D)**, **(E–H)** and **(I–L)**. miR-21 was detected using TSA-Cy5 substrate (white) and HER2 with Cy3-conjugated anti-rabbit antibody (red). All sections were counterstained with DAPI (blue). miR-21 ISH signal is seen in HER2-positive cancer cells in cases **(A–D)** and **(E–H)** [examples are indicated by arrows in **(A–D)**], whereas HER2-positive cancer cells in case **(I–L)** are miR-21-negative. The examples are from tumors in the reference group and are representative for the three tumors. Bars: 20 μm.

In order to perform double fluorescence analysis of HER2 and miR-21, we employed a polyclonal antibody, which was compatible with the proteinase-K-directed antigen retrieval needed for miR ISH on FFPE samples. Six samples from the reference group were selected for miR-21/HER2 double immunofluorescence based on (1) the intense staining for HER2 with the polyclonal antibody and (2) the miR-21^CC^ and/or miR-21^St^ positive staining. As expected, we found miR-21 ISH signal in HER2-positive cancer cells (Figure 3). We noted that the miR-21 staining intensity in HER2-positive cancer cells varied from absent to strongly positive. For comparison, in a case with prevalent stromal miR-21, we found miR-21 positive stromal cells surrounding HER2-positive clusters of cancers cells (Figure 3).

### MicroRNA-21 staining patterns and recurrence after HER2-directed therapy

**Figure 4:**
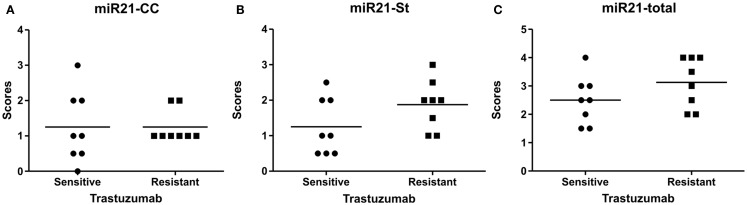
**MicroRNA-21 scores in the HER2 study group**. The miR-21 scores for cancer cell-associated miR-21 expression, miR-21^CC^ **(A)**, miR-21 stromal expression, miR-21^St^ **(B)**, and the total summarized miR-21 scores **(C)**. The scores are averages of two observers and here presented in scatter grams. Mann–Whitney *U* test: *p* = 0.69 for miR-21^CC^, *p* = 0.13 for miR-21^St^, and *p* = 0.20 for total miR-21.

MicroRNA-21 ISH was then performed in parallel on the 16 HER2-positive cases with known disease status after adjuvant trastuzumab. Eight of the patients experienced no recurrent disease after treatment within the 5–8 years follow-up period (sensitive tumors), whereas the other eight patients experienced recurrent disease (resistant tumors). Examination of the miR-21 staining patterns in these samples indicated expression patterns similar to those observed in the *reference group*, thus both tumors with predominant miR-21^CC^ and miR-21^St^ were present (Figure 4). However, there was no significant difference in the miR-21^CC^ and miR-21^St^ as well as in the summarized total average miR-21 scores, when comparing resistant and sensitive tumors (Mann–Whitney *U* test). Thus, we found no indications that miR-21 expression patterns could help to identify HER2-positive patients resistant to adjuvant trastuzumab in this study group.

## Discussion

MicroRNA-21 is one of the most prevalent miRs in solid tumors, and increased expression levels are associated with poor prognosis both in untreated early-stage colon cancer patients (8, 10, 15) and in breast and colon cancer patients treated with chemotherapy (8, 9, 36, 37). In addition, several recent studies have suggested that miR-21 confers drug resistance to cancer cells *in vitro* (38, 39), including resistance to trastuzumab treatment of HER2-positive patients (19). We assumed that if miR-21 indeed confers drug resistance in HER2 breast cancer patients, knowing the specific cellular origin of miR-21 in the tumors may help to predict response to HER2-directed therapy. In this study, we show that miR-21 is highly expressed in high-grade breast cancers, both in HER2-positive and negative cases, and that miR-21 ISH signal can be seen in both stromal cells and cancer cells in varying expression levels.

Gong et al. (19) found increased miR-21 ISH signal in resistant tumors before and after trastuzumab-directed therapy, and showed that miR-21 antisense oligonucleotides could restore trastuzumab sensitivity in resistant cells by inducing PTEN expression, suggesting that miR-21 mediates trastuzumab resistance and that antagonizing miR-21 in a therapeutic setting could sensitize cancer cells to HER2-directed therapy. Indeed, we found that both miR-21 ISH signal and HER2 immunoreactivity are seen in the same cancer cells, which would be a prerequisite for successful combination of anti-miR-21 and HER2-directed therapy. In order to assess if miR-21^CC^ expression could predict the response to trastuzumab therapy, we examined the cellular expression of miR-21^CC^ in 16 HER2-positive tumors, half of which relapsed within the 5–8 years follow-up period after adjuvant trastuzumab. However, since we also found miR-21^CC^ expression in trastuzumab-sensitive tumors at the same frequency, our findings do not indicate that high miR-21^CC^ can predict recurrence. Based on our studies of 16 patients treated in the adjuvant setting, our observations imply that miR-21 did not confer trastuzumab resistance, a finding which appears inconsistent with the conclusion drawn by Gong et al. (19), who investigated 32 patients treated in the neoadjuvant setting. However, the major difference in treatment protocols of the two studies does not justify a direct comparison of the results.

Although the relatively small sample size offered little statistical strength to our observations, it should be noted that the concomitant adjuvant chemotherapy may have been a confounding factor that prevented identification of a direct link between miR-21 expression and trastuzumab resistance. Likewise, small patient groups will be relatively sensitive to genetic variation, which in our case may also have contributed to cover the impact of miR-21 as a predictive biomarker. Another parameter that may have prevented identification of a direct link between miR-21 and trastuzumab resistance is the semiquantitative scoring approach employed. In order to minimize experimental variation in staining intensities, all sections, both in the reference group and in the study group, were processed identically and stained in parallel on an automated platform. Manual scoring was then performed on digital whole slides by two observers, and since considerable variation between samples could easily be discriminated, we assumed that our scoring approach was not a limiting factor. Furthermore, a manual scoring approach could potentially be implemented in the daily diagnosis. In previous studies, we used image analysis to obtain quantitative estimates of miR-21 expression (10, 12, 15), but common for those studies, the expression patterns were more homogenous. Here, the cellular origin of expression was a key parameter. It cannot be excluded that alternative semiquantitative approaches may have classified the cases better. For example, RT-qPCR analyses of microdissected tissue compartments, stroma and cancer cells, may potentially provide useful quantitative estimates of the miR-21 expression. Thus, more studies, including examination of the primary and recurrent tumors in both the adjuvant and neoadjuvant settings, are needed to better address how the miR-21^CC^ (and miR-21^St^) parameters potentially can be used as predictive biomarkers.

In our previous study of miR-21 in grade 1–2 breast cancers (12), none of the 24 included cases showed noteworthy expression in the cancer cells. In the current study, 9 of the 36 cases (25%) showed intense miR-21^CC^ (miR-21^CC^ score 2 or above). These miR-21^CC^ cases included both hormone receptor-positive and -negative cases. Furthermore, miR-21^CC^ did not correlate with any of the other clinical parameters analyzed in the HER2-positive tumors. In HER2-negative tumors, we found a significant positive correlation between miR-21^CC^ and PR. The significance of this association will need to be further explored. Because miR-21 was found strongly expressed in myoepithelial cells surrounding occasional CIS structures in some of the lesions, it is tempting to speculate whether the miR-21^CC^ cases were all of the basal subtype. However, this is unlikely since some of the miR-21^CC^ cases were ER-positive. Thus, our data suggest that the miR-21^CC^ lesions represent an independent group of grade 3 breast cancers. The apparent variation in the cellular expression pattern of miR-21 is an important feature that may impact on targeted therapy since miR-21 in different cell populations may have different (mRNA) targets. Sempere et al. (40) analyzed breast cancers of various grades and hormone receptor status and reported frequent expression in cancer cells as well as expression in the breast cancer-associated fibroblast in some cases. More studies are needed to better establish whether high miR-21 expression in cancer cells is confined to grade 3 breast cancers.

As mentioned above, increased miR-21 expression levels are associated with adverse prognosis in several types of cancer. Experimental studies of tumor models in mice support these findings. In a K-ras-dependent lung cancer model, overexpression of miR-21 caused increased tumor formation, whereas miR-21 deficiency reduced the tumor formation (41). In a skin carcinogenesis model, miR-21 deficiency resulted in reduced papilloma formation (42). In addition, mice lacking the tumor suppressor p53 showed reduced tumor incidence if also lacking miR-21 (43). Thus, based on both clinical and experimental findings, miR-21 is likely a positive driver in the oncogenic process.

Two of the best described miR-21 targets are the tumor suppressors PDCD4 and PTEN. In the context of this study, it is notable that Huang et al. (44) found that activation of HER2 up-regulates miR-21 in breast cancer cell lines and causes successive down-regulation of PDCD4 preventing the tumor cells to enter apoptosis. However, PDCD4 is likely a multifunctional protein, whose role in cancer is only partly understood. PDCD4 was originally found as a nuclear antigen of proliferating cells (45), while others found PDCD4 associated with apoptosis (46, 47). In addition, PDCD4 was reported to be an inhibitor of neoplastic transformation and metastasis (48, 49). In breast cancer tissue, PDCD4 is seen in both normal and malignant epithelial cells and localizes to the nuclei and/or the cytoplasm (50). It remains to be established if miR-21 is involved in the regulation of all of these pivotal functions of PDCD4. Like PDCD4, PTEN has been found in relation to cell proliferation and apoptosis (51), and its presence has been linked to drug resistance (30). Lack of PTEN in breast tumor stroma has, in model systems, been found to strongly enhance transformation of the mammary epithelium (52, 53). In our study, high levels of miR-21 in the breast cancer stroma, which would cause miR-21-directed loss of stromal PTEN, were weakly (*p* = 0.13) associated with resistance to adjuvant trastuzumab. Trastuzumab resistance was reported in patients with breast cancers that were PTEN-negative in immunohistochemistry (30), however, a systematic delineation of the presence of PTEN in different cellular compartments in breast cancer lesions is highly warranted, particularly in the light of recent studies implying that drug resistance can be generated through tumor stroma (31–34).

In our previous study of grade 1 and 2 breast cancers (12), we found that high stromal miR-21 levels determined by image analysis correlated significantly with increased Ki67 proliferation index. Quantitative assessment by image analysis of the miR-21 ISH signal in the current grade 3 samples was not accomplished due to the complex expression patterns as discussed above. However, our analysis of the miR-21 expression scores in the stroma or cancer cells in the current grade 3 cancers did not reveal correlation with the Ki67 index.

In conclusion, we have shown that the miR-21 expression patterns in HER2-positive breast cancers are highly variable being present in cancer and/or stromal cells, and not linked to known clinical parameters. In the relatively small group of HER2-positive tumors studied, our miR-21 ISH analyses did not contribute significantly to the identification of patients with recurrent disease after adjuvant trastuzumab. We noted that both miR-21^CC^ and miR-21^St^ were independent of ER, PR, Ki67, and p53, suggesting that more extensive studies on trastuzumab resistance are warranted across additional subgroups of breast cancer. Molecular markers of trastuzumab resistance like the serum-derived inflammatory biomarkers (34), or expression of PTEN (30), or truncated HER2 (54) have so far not successfully showed to be clinically valuable predictive biomarkers.

## Conflict of Interest Statement

The authors declare that the research was conducted in the absence of any commercial or financial relationships that could be construed as a potential conflict of interest.
